# Aesthetic Efficacy and Safety of Combined Microfocused Ultrasound With Visualization and Calcium Hydroxylapatite Treatment: A Systematic Review of Human Evidence

**DOI:** 10.1093/asj/sjae239

**Published:** 2025-01-30

**Authors:** Mojgan Amiri, Renald Meçani, Christa D Niehot, Guido Muharremi, Julieta Spada, Rossana Vasconcelos, Tatjana Pavicic, Sonja Sattler, Melissa K Levin, Siew Tuck Wah, Julia Carroll, Sonya Cook, Taulant Muka

## Abstract

Although microfocused ultrasound with visualization (MFU-V) and calcium hydroxylapatite– carboxymethylcellulose (CaHA-CMC) have their individual strengths and have demonstrated effectiveness in aesthetic improvement and improving skin laxity, a combined treatment may sometimes be required to achieve comprehensive aesthetic enhancements that meet patients’ needs and preferences. This review systematically summarizes the available evidence on combined MFU-V and CaHA-CMC treatment. A comprehensive search was conducted in Embase, MEDLINE ALL (Ovid), Web of Science Core Collection, and Cochrane Central. We included studies conducted in adults that examined the effectiveness, safety, and/or mechanism of action of combined MFU-V and CaHA-CMC treatment. Out of 4019 references, 11 studies, mainly pre-post studies, were included in this analysis. Overall, regardless of the area treated, improvements in global aesthetic scales, skin quality parameters, and patients’ satisfaction following combined treatment, accompanied by mild to moderate adverse effects, were found. In addition, histological studies indicated increased neocollagenesis and elastin synthesis posttreatment. Our review highlights promising outcomes from combined MFU-V and CaHA-CMC treatment. Nevertheless, due to the limited number of studies, further research is essential to gain a deeper understanding of this combined treatment's efficacy, safety, and applicability.

**Level of Evidence: 3:**

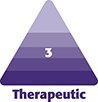

See the Commentary on this article here.

Calcium hydroxylapatite–carboxymethylcellulose (CaHA-CMC) is known as a regenerative biostimulator^1^ and has emerged as a prevalent choice for cosmetic procedures. Additionally, microfocused ultrasound with visualization (MFU-V), an energy-based treatment, is widely acknowledged as the gold standard for nonsurgical skin lifting and tightening.^2,3^

CaHA-CMC and MFU-V represent distinct modalities for skin rejuvenation. The skin regenerative properties linked with CaHA-CMC involve several mechanisms and factors, including its role in promoting cell proliferation, collagen and elastin synthesis, angiogenesis, and proteoglycan formation.^4^ Meanwhile, MFU-V operates by generating controlled thermal coagulation zones within specific depths of the skin without damaging the epidermis and stratum corneum.^5,6^ This process is believed to result in contraction of denatured collagen and initiate collagen remodeling and neocollagenesis.^5^

Studies have shown enhancements in aesthetic improvement scores after the injection of CaHA-CMC with high patient satisfaction rates.^7^ Similarly, a recent meta-analysis from our group found that MFU-V treatment led to an aesthetic improvement of over 80% and an overall patient satisfaction rate of 84%, with a moderate pain score of 4.87 and mild to moderate adverse events reported.^8^ However, addressing multiple aging skin concerns often necessitates combining different aesthetic treatments.^9-11^ Given the unique strengths and limitations of each modality, combining products may be essential to achieve optimal outcomes. Although multiple studies have explored the combination of CaHA-CMC and MFU-V on aesthetic outcomes, there has been no systematic appraisal of evidence on understanding the efficacy, safety, and mechanism of action of combining these 2 treatments.

In the current study, we conducted a systematic review of the existing literature, exploring the aesthetic effectiveness, patient satisfaction levels, improvements in skin quality parameters, and safety profiles as well as potential mechanisms of action associated with the combination of MFU-V and CaHA-CMC.

## METHODS

This systematic review adhered to recent guidelines and the PRISMA reporting standards.^12,13^ The study protocol was registered in the OSF Registries on August 4, 2023.

### Data Sources and Search Strategy

An expert librarian (C.D.N.) developed the search strategy; Embase, MEDLINE ALL (Ovid), Web of Science Core Collection, and Cochrane Central were explored from study inception up to August 20, 2023. Furthermore, the first 200 results from Google Scholar were imported. The details of the search strategy and keywords are presented in Supplemental Table 1. To further identify relevant studies, the reference lists of the final included studies were manually reviewed.

### Eligibility Criteria

The inclusion criteria were as follows: (1) published interventional and observational studies in adults (≥18 years old); (2) investigating combined treatment with MFU-V and CaHA-CMC; (3) treatments administered either immediately after each other or with a time interval; (4) outcome related to aesthetics, skin aging, skin quality, patient satisfaction, safety, or mechanism of action; (5) irrespective of the participants’ characteristics and health status, the area treated, and the sample size. Reviews, letters to editors, conference abstracts, and studies conducted on animals, children, or adolescents were excluded.

### Study Selection and Data Extraction

Retrieved references and full texts were independently screened (by M.A., R.M., and G.M.) in duplicate based on the eligibility criteria. Data from the included studies were extracted (by M.A. and R.M.) based on a predesigned form. The extracted information included the first author's name, study design, publication year, location, number of participants, sex distribution of the population, participants’ health status/characteristic at study entry, age, follow-up duration, ethnicity, skin type, treated area, device name and brand, transducer information, dermal filler/injectable brand, dilution and dosage, treated depth, outcomes assessment methods, adjustments, and any measure of frequency or association. Any disagreements were resolved through discussions with a third researcher (T.M.).

### Quality Assessment

The quality of included studies was evaluated using the Cochrane Collaboration Risk-of-Bias 2 tool for nonrandomized studies, including observational and pre-post interventional studies^14^ or the National Institutes of Health quality assessment tool for case series studies.^15^

### Statistical Analysis

Due to the limited number of studies, diversity in participant characteristics, outcomes, and measurement methods, we performed a qualitative review. We present the size, direction of change, and statistical significance of the observed changes in each study and created tables to outline the study characteristics, their findings, and an evaluation of the study methodologies.

## RESULTS

### Eligible Studies and Study Characteristics

**Figure 1.**
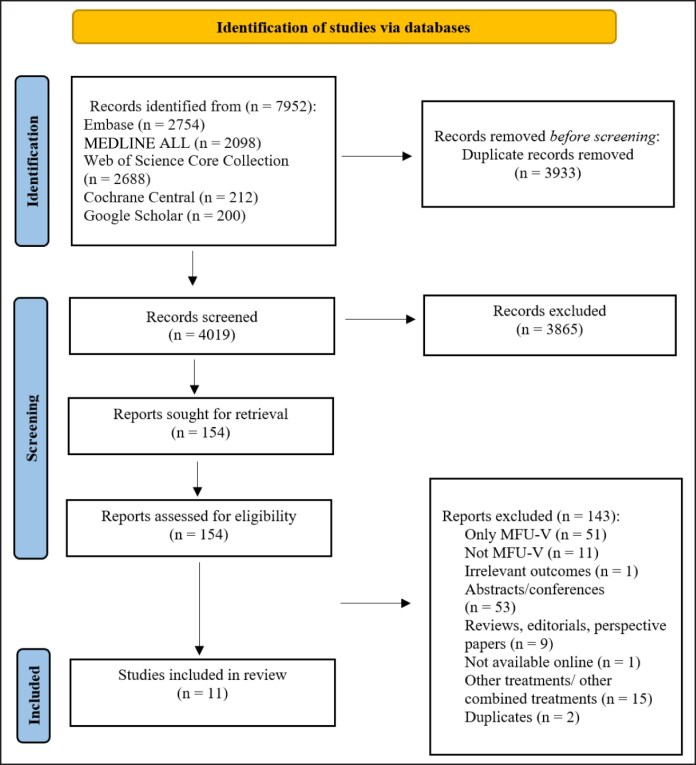
Flowchart of the identification, screening, eligibility, inclusion, and exclusion of the retrieved studies.

Out of 4019 retrieved references, 11 studies were included in the current systematic review (Figure 1). The included studies were published between 2014 and 2023, with 6 designed as pre-post interventional,^16-21^ 2 retrospective observational studies,^22,23^ 1 prospective observational study,^24^ and 1 case series.^25^ Among these studies, 2 reported histological findings^17,22^ and 1 included a comparison group for a subset of outcomes.^21^ One study only reported histological findings.^26^ Four studies were conducted in Brazil,^16,22,23,26^ 2 in Singapore,^18,20^ 2 in the United States,^19,21^ and 1 each in Spain,^25^ Russia,^17^ and Germany.^24^ Treated regions included the face, neck, décolletage, abdomen, thigh, buttock, and knee. The number of study participants ranged between 1 to 60. In most studies, the MFU-V was administered first, followed by the application of CaHA-CMC. In 2 studies, the sequence was reversed, with CaHA-CMC injected first followed by MFU-V administration.^17,26^ The administrations of MFU-V and CaHA-CMC were 8 months and 12 weeks apart in 2 studies,^17,24^ while they were administered during the same treatment session in the remaining 9 studies. Most studies employed a single-session treatment for each modality, but 2 studies employed 2 sessions of CaHA-CMC^17,25^ (Supplemental Table 2). All studies were rated as having a serious risk of bias for confounding, with the majority also receiving a serious risk-of-bias rating for outcome measurement (Supplemental Table 3). The only case series study received a fair quality assessment score (Supplemental Table 4).

## RESULTS

### Aesthetic Effectiveness

Aesthetic effectiveness scores, skin quality parameters, and patients’ satisfaction were reported by 10 studies across various body parts (Supplemental Table 5). A study on jawline treatments reported that the proportion of subjects showing an improvement of at least 1 point increased with combined treatment (up to 89% by week 48), with increased skin thickness at week 24 and improved firmness at week 48 compared with baseline (*P* < .05).^24^ Significant improvements in scar severity (*P* = .002) and high patient satisfaction were noted following combined treatment by another study in facial area.^16^ A study on the neck and décolletage regions showed improved neckline score (*P* < .001) and décolletage score (*P* < .001), and high satisfaction levels (*P* < .001) vs baseline.^23^ In another study, treatment across face, neck, and décolleté regions resulted in better age-related aesthetics and global improvements, such as significant improvements in marionette lines score, jawline contour score, and neck score at Month 15 (*P* < .05).^17^ Brachial treatment improved the arm visual analog scale score (*P* < .05) and improved skin quality with reduced laxity and increased firmness throughout the course of the study (*P* < .05 vs baseline), with patients being satisfied with the results.^18^ A study on outer thigh treatment showed enhanced body image (*P* < .01) and reduced distress related to excess skin (*P* < .01), with high satisfaction levels (*P* < .01) vs baseline.^19^ An investigation of the combined treatment on buttocks and thigh revealed improvements in skin laxity and cellulite severity, with 50% of participants very satisfied, and 45% satisfied.^22^ In addition, a case series study reported improvement in laxity score in the chest and buttocks.^25^ Treatment across abdominal, back, thigh, and leg regions led to improvements in striae distensae albae severity over time (*P* < .05) and overall aesthetic improvement, with all patients satisfied or very satisfied.^20^ A study evaluating the effect of combined treatment on the area of the knee showed mild to moderate improvements in aesthetic appearance and patient satisfaction over time, and no significant effect on skin roughness index and rhytid depth was observed.^21^

### Histological Findings

Histological findings were reported by 3 studies, each on 1 sample (Supplemental Table 6). Yutskovskaya et al observed a significant increase in collagen Type I levels following CaHA-CMC injection compared with baseline (mean [standard deviation], 0.9 [0.5]-2.4 [0.3] points).^17^ This increased to 4.4 [0.3] points after combined treatment (*P* < .05). In this study, biopsies at Month 12, 4 months after administration of MFU-V and 8 months after CaHA-CMC, were used to evaluate the effects of combined CaHA-CMC and MFU-V treatments. Similarly, collagen Type III levels significantly increased after CaHA-CMC injection (1.5 [0.2]-3.5 [0.3] points), reaching 5.7 [0.2] points after combined treatment (*P* < .05). Additionally, this study noted significant increases in angiogenesis, elastogenesis, and CD34 expression, an endothelial marker for newly formed blood vessels, post–CaHA-CMC injection, which were further enhanced after MFU-V treatment compared with baseline (*P* < .05). The proliferation index Ki67 also increased post–CaHA-CMC injection compared with baseline (*P* < .05), with a subsequent relative decrease following MFU-V treatment (*P* > .05). In 2 other studies, neocollagenesis following combined treatment was the primary focus. Casabona et al reported thicker and denser collagen fibers in the combined treatment site compared with MFU-V alone, indicating an enhancement in the number and quality of collagen and elastin fibers.^26^ The final study aimed to assess the influence of different CaHA-CMC dilutions, including 1:0.16, 1:0.3, 1:0.6, 1:1, 1:2, and 1:6.5, compared with untreated skin, and whether MFU-V enhanced the CaHA-CMC–induced neocollagenesis. A peak in neocollagenesis was observed 90 days posttreatment in samples treated with a 1:1 CaHA-CMC dilution, whether alone or combined with MFU-V. When CaHA-CMC was combined with MFU-V, the 1:1 dilution led to a 251% increase in collagen Type III fibers compared with control tissue. Moreover, the highest conversion of collagen Type III to Type I occurred in samples injected with 1:1 (103%) and 1:0.6 (93%) CaHA-CMC dilutions without subsequent MFU-V treatment, compared with untreated control tissue. Following combined treatment, conversion to Type I collagen increased by up to 41%.^22^

### Safety

Nine of the included studies reported adverse events following combined treatment. Procedures were generally well-tolerated by patients, with no or minor adverse events reported. No serious adverse events were reported (Supplemental Table 7). Commonly reported adverse events included erythema, swelling, bruising, and pain/discomfort, all of which were mild in severity and resolved during subsequent visits in the studies.^16-24^

## DISCUSSION

This review has summarized available evidence on combined treatment with MFU-V and CaHA-CMC. Overall, the combined MFU-V and CaHA-CMC treatment demonstrated favorable outcomes in improving aesthetic effectiveness, enhancing skin quality parameters, and increasing patient satisfaction across various treated regions with no serious adverse events. In addition, histological findings suggest increased neocollagenesis and elastin synthesis following the combined treatment.

As the demand for nonsurgical aesthetic procedures in facial and nonfacial areas continues to increase, various interventions, such as injectable CaHA-CMC (Radiesse, Merz Aesthetics, Raleigh, NC) and MFU-V (Ultherapy, Merz Aesthetics, Raleigh, NC), have attracted significant attention. CaHA-CMC is approved for correcting facial wrinkles and folds, treating facial lipoatrophy in human immunodeficiency virus patients, enhancing jawline contours, restoring volume to the hands, and improving décolletage. MFU-V is cleared for noninvasively lifting brow, submental, and neck tissues, as well as reducing décolletage wrinkles.^27^

The tissue regeneration potential due to the heat generated from MFU-V and the spherical structures of CaHA acting as a scaffold^28^ may mutually enhance each other’s effectiveness by promoting cellular activity and the production of extracellular matrix proteins at the treatment site. Studies have shown that controlled heat stress, produced by energy-based devices such as various laser therapies, can increase the expression of heat-shock proteins, which may contribute to enhanced tissue regeneration.^29-33^ Because MFU-V is an energy-based device that raises the temperature in targeted skin areas at specific depths, it is expected to operate through a similar mechanism, although the current evidence specific to MFU-V remains to be demonstrated.

In practice, CaHA-CMC and MFU-V are both recognized as aesthetic collagen stimulation procedures. Research indicates that CaHA-CMC can promote cell proliferation, collagen synthesis, angiogenesis, and elastin formation, suggesting regenerative properties.^4^ Similarly, studies have suggested neocollagenesis after MFU-V through the creation of thermal coagulation zones.^6,26,34^ Based on the available literature, combining MFU-V and CaHA-CMC treatments appears to enhance neocollagenesis compared with the use of CaHA-CMC^17,22^ or MFU-V alone.^26^ These findings are supported by improvements seen in aesthetic scales and skin quality parameters, such as increased skin thickness and firmness, suggesting that the synergistic effects of MFU-V and CaHA-CMC can lead to favorable outcomes in aesthetic procedures.

To our knowledge, this is the first systematic review addressing combined treatment with MFU-V and CaHA-CMC. We implemented a comprehensive search strategy to ensure the inclusion of the most relevant references. However, our study reveals several limitations among available studies. A very limited number of studies have investigated the effects of the combined treatment, resulting in inadequate exploration of both facial and nonfacial regions. Most studies lack control groups, limiting the ability to compare treatment efficacy. Additionally, small sample sizes affect the statistical power and reliability of the findings. Histological conclusions are drawn from single samples per study which might potentially weaken the robustness of the findings. Different ethnicities, groups, and genders are not represented in the included studies, limiting our understanding of treatment effects across different racial/ethnic groups and among men.

Future studies should address existing gaps in the literature by conducting well-designed randomized controlled trials with larger sample sizes. Comparing combined treatments with CaHA-CMC and/or MFU-V monotherapy will enhance our understanding of their aesthetic effectiveness. Additional research is needed in both facial and nonfacial areas to determine where these combinations are most effective. Studies should also include diverse populations to assess variations in treatment outcomes and examine the impact of factors such as age, skin type, and health status. Furthermore, future research is needed to understand which patients can benefit the most from the MFU-V and CaHA-CMC combined treatment because the indications for patients to receive the combination therapy varied in the available studies and the impact of combined therapy might be different in patients with different characteristics. For example, Kerscher et al suggested combined therapy might be an option for subjects aged 45 years or older with a higher grade of extrinsic skin aging and sagging, and relatively low skin firmness and elasticity.^24^ Additionally, the importance of timing in combining treatments to achieve optimal results is crucial. Although most available studies applied the treatments during the same treatment session, some studies incorporated time intervals. Also, there is no consensus on the ideal interval between treatments when they are not performed on the same day; however, a 12-week gap for the follow-up procedure has been suggested and it is recommended to apply the MFU-V first when treatments are given on the same day and in the same area. Among the included studies, most treatments were initiated with MFU-V, while others started with CaHA-CMC injection. Therefore, analyzing the impact of treatment timing and sequence on aesthetic outcomes would be valuable for enhancing our understanding of the optimal treatment protocol.

## CONCLUSIONS

Our systematic review has synthesized the available evidence on combined treatment with MFU-V and CaHA-CMC, revealing promising outcomes in aesthetic effectiveness, patient satisfaction following treatment, and skin quality improvement across various treated regions, along with observed collagen-stimulating effects and the absence of serious adverse events. However, although our review sheds light on these promising outcomes, there is a need for further research. By addressing the identified research gaps and methodological considerations, further investigations can contribute to deepening our understanding and optimizing the efficacy, safety, and applicability of combined aesthetic procedures.

## Supplemental Material

This article contains supplemental material located online at https://doi.org/10.1093/asj/sjae239.

## Supplementary Material

sjae239_Supplementary_Data
