# Supplementary material for: Aesthetic Efficacy and Safety of Combined Microfocused Ultrasound With Visualization and Calcium Hydroxylapatite Treatment: A Systematic Review of Human Evidence
Source: Aesthet Surg J. 2025 Jan 30;45(6):638–42. doi: 10.1093/asj/sjae239 (PMC12080884; doi:10.1093/asj/sjae239)
Supplement: sjae239_Supplementary_Data [file sjae239_supplementary_data.docx]

**Supplemental Tables: Aesthetic Efficacy and Safety of Combined Microfocused Ultrasound with Visualization and Calcium Hydroxylapatite Treatment: A Systematic Review of Human Evidence**

**Supplemental Table 1.** Designed search strategies.

| Database | Strategy |
| --- | --- |
| Embase | ('microfocused ultrasound'/exp OR 'microfocused ultrasound with visualization'/exp OR 'ultherapy'/exp OR 'high intensity focused ultrasound'/exp OR (((focused* OR microfocused) NEAR/3 (ultrasound*)) OR ultherap* OR MFU-V OR MFUV OR IFUS OR HIFU):ab,ti,kw) AND ('body regions'/exp OR 'skin'/exp OR 'face'/exp OR 'hand'/exp OR (skin OR derm* OR epiderm* OR dermal* OR wrinkle* OR face OR facial OR hand OR hands OR cheek* OR buttock* OR jaw* OR neck OR elbow* OR foot OR feet OR decolle* OR arm OR arms OR leg OR legs OR forehead* OR ear OR ear OR fold OR folds OR line OR lines OR chin OR eyelid* OR lip OR lips OR abdom* OR nonfacial* OR tigh OR tighs OR chest OR chests OR orbit* OR periorbit* OR submentum OR submental OR brow OR brows):ab,ti,kw) NOT ([Conference Abstract]/lim OR [preprint]/lim) |
| Medline | ("High-Intensity Focused Ultrasound Ablation"/ OR (((focused* OR microfocused) ADJ3 (ultrasound*)) OR ultherap* OR MFU-V OR MFUV OR IFUS OR HIFU).ab,ti,kf.) **AND** (exp "Body Regions"/ OR exp "Skin"/ OR (skin OR dermis OR epiderm* OR derm* OR wrinkle* OR face OR facial OR hand OR hands OR cheek* OR buttock* OR jaw* OR neck OR elbow* OR foot OR feet OR decolle* OR arm OR arms OR leg OR legs OR forehead* OR ear OR ear OR fold OR folds OR line OR lines OR chin OR eyelid* OR lip OR lips OR abdomen* OR abdom* OR nonfacial* OR tigh OR tighs OR chest OR chests OR orbit* OR periorbit* OR submentum OR submental OR brow OR brows).ab,ti,kf.) NOT (news OR congres* OR abstract* OR book* OR chapter* OR dissertation abstract*).pt. |
| Web of Science | TS=(((((focused* OR microfocused) NEAR/2 (ultrasound*)) OR ultherap* OR MFU-V OR MFUV OR IFUS OR HIFU)) AND ((skin OR derm* OR epiderm* OR dermal* OR wrinkle* OR face OR facial OR hand OR hands OR cheek* OR buttock* OR jaw* OR neck OR elbow* OR foot OR feet OR decolle* OR arm OR arms OR leg OR legs OR forehead* OR ear OR ear OR fold OR folds OR line OR lines OR chin OR eyelid* OR lip OR lips OR abdom* OR nonfacial* OR tigh OR tighs OR chest OR chests OR orbit* OR periorbit* OR submentum OR submental OR brow OR brows))) **AND** DT=(Article OR Review OR Letter OR Early Access) |
| Cochrane | ((((focused* OR microfocused) NEAR/3 (ultrasound*)) OR ultherap* OR MFU NEXT/1 V OR MFUV OR IFUS OR HIFU):ab,ti,kw) **AND** ((skin OR derm* OR epiderm* OR dermal* OR wrinkle* OR face OR facial OR hand OR hands OR cheek* OR buttock* OR jaw* OR neck OR elbow* OR foot OR feet OR decolle* OR arm OR arms OR leg OR legs OR forehead* OR ear OR ear OR fold OR folds OR line OR lines OR chin OR eyelid* OR lip OR lips OR abdom* OR nonfacial* OR tigh OR tighs OR chest OR chests OR orbit* OR periorbit* OR submentum OR submental OR brow OR brows):ab,ti,kw) |
| Google Scholar | 'focused\|microfocused ultrasound'\|ultherapy skin\|dermal\|dermis\|wrinkle\|face\|nonfacial\|hand\|cheek\|buttock\|neck\|arm\|tigh\|chest\|decolletage\|abdomen\|eyelid\|lip\|chest\|brow |

**Supplemental Table 2.** Characteristics of the included studies evaluating the combined MFU-V and CaHA-CMC treatment.

| **Author, year** | **Country** | **Design** | **Follow-up** | **No. participants^1^** | **Sex (%F)** | **Health status/ characteristics** | **Age^1^** | **Ethnicity** | **Treated cites** | **Treatment protocol** | **MFU-V device / CaHA brand** | **Outcomes** |
| --- | --- | --- | --- | --- | --- | --- | --- | --- | --- | --- | --- | --- |
| Kerscher, 2019 ^1^ | Germany | Observational (Prospective) | 48 w | MFU-V only: 22  Combined: 9 | F (100%) | With moderate-to-severe skin laxity in the submental region and a body mass index of 20–30 kg/m². | 52.32±9.31^*^ | NM | Jawline | **MFU-V:** A single treatment using two transducers beginning with a 4-MHz, 4.5-mm-depth transducer delivering 350 lines in total, followed by a 7-MHz, 3.0-mm-depth transducer delivering a total of 270 lines..  **CaHA:** Subjects who did not show an improvement of ≥ 1 point on the Merz Aesthetic Scale for the lower face after 12 weeks, were additionally treated with CaHA to the jawline, 1.5 mL on each side of the face. | **MFU-V:** Ulthera® System (Ulthera, Inc., Raleigh, NC, USA)  **CaHA:** Radiesse® (Merz North America, Inc., Raleigh, NC, USA) | - Clinician Improvement Scale,  - Clinician Global Aesthetic  Improvement Scale,  - Skin laxity and jawline contour,  - Skin thickness and firmness,  - Skin hydration and PH,  - Adverse events. |
| Casabona, 2017 ^2^ | Brazil | Interventional (Pre-Post) | 90 d | 10 | B (NM) | With moderate-to-severe facial acne scars. | 37.6±10 | NM | Both sides of the face | **MFU-V:** Two transducers at focal depths of 3.0 mm and 1.5 mm with a total of 75 treatment lines.  **CaHA-CMC:** Immediately after MFU-V treatment. Same acne scars were injected with 1.5 mL of CaHA-CMC filler diluted 1:1 with 1.5 mL of lidocaine 2%. One-half of the syringe was used to inject each side of the face. | **MFU-V:** Ultherapy (Brand was not mentioned).  **CaHA-CMC:** Radiesse® (Brand was not mentioned). | - Scar severity,  - Patients’ satisfaction,  - Adverse events. |
| Casabona, 2017 ^3^ | Brazil | Observational (Retrospective) | 90 d | 47  (Neck n=29), decolletage (n=5), both areas (n=13)) | B (93.6) | With moderate-to-severe lines on the neck and/or decolletage. | 49.3±9.9 | NM | Neck and/or decolletage | **MFU-V:** A single treatment using a 7-MHz transducer at a focal depth of 3.0 mm and a 10 MHz transducer at a depth of 1.5 mm, applying 150 lines per site.  **CaHA-CMC:** Immediately after MFU-V treatment. Treatment received with CaHA-CMC 1.5 mL diluted 1:1 with 1.5 mL of 2% lidocaine solution.  For the neck, CaHA-CMC was injected subdermally. | **MFU-V:** Ulthera® System (Ulthera, Inc., Raleigh, NC, USA)  **CaHA-CMC:** Radiesse® (Merz North America, Inc., Raleigh, NC, USA) | - Neckline score,  - Decolletage score,  - Patients’ satisfaction,  - Adverse events and pain. |
| Yutskovskaya, 2020 ^4^ | Russia | Interventional (Pre-Post) | 15 m | 19 | F (100) | Healthy with body mass index < 21 kg/m^2^. | 30-45 | NM | Face, neck, décolleté | **CaHA-CMC:** Two treatment sessions (Right side: day 1 and month 4; Left side: month 12 and month 15). Radiesse 1.5 mL and Radiesse 0.8 mL were used.  **MFU-V:** A single treatment at month 8 using a 7 MHz at focal depth of 3.0 mm and 4 MHz at focal depth of 4.5 mm, providing 1200 pulses in total. | **CaHA-CMC:** Radiesse® (Merz North America, Inc., Raleigh, NC, USA)  **MFU-V:** Ulthera® System (Ulthera, Inc., Raleigh, NC, USA) | - Clinician age-related aesthetics improvement,  - Changed volumes of the submental area and cervicomandibular angle,  - Clinician and patients global aesthetic improvement,  - Adverse events and pain. |
|  |  | Histological findings | 12 m | 1 | F | Healthy with body mass index < 21 kg/m^2^. | NM | NM | Lower abdominal quadrant | **CaHA-CMC:** Two treatment sessions at day 1 and month 4. Radiesse 1.5 mL and Radiesse 0.8 mL were used.  **MFU-V:** A single treatment at month 8 using a 7 MHz at focal depth of 3.0 mm and 4 MHz at focal depth of 4.5 mm, providing 1200 pulses in total. | **CaHA-CMC:** Radiesse® (Merz North America, Inc., Raleigh, NC, USA)  **MFU-V:** Ulthera® System (Ulthera, Inc., Raleigh, NC, USA) | - Neocollagenesis, CD34 and other histomorphological features. |
| Ramirez, 2021 ^5^ | Singapore | Interventional (Pre-Post) | 24 w | 12 | F (100) | With skin laxity in the brachial regions and mass index < 28 kg/m^2^. | 51.3±7.3 | Asian, Caucasian | Brachial regions | **MFU-V:** A single treatment with two depths using the 4.0 MHz- 4.5 mm depth transducer for deeper penetration to the superficial fascial layer in the first pass, followed by the 7.0 MHz- 3.0 mm depth transducer for more superficial penetration to the deep dermis in the second pass, delivering a total of 180–240 treatment lines were per each arm using each transducer. For a total of 360– 480 lines per arm with both transducers, with more lines for patients with larger arm volumes.  **CaHA-CMC:** Two different dilutions were used depending on investigator's assessment of skin thickness. A dilution ratio of 1:2 (1.5 ml of CaHA-CMC diluted with 0.3 ml 2% lidocaine and 2.7 ml normal saline, for a total volume of 4.5 ml per arm) was used for subjects with thinner skin. A dilution ratio of 1:1 (1.5 ml of CaHA-CMC diluted with 0.3 ml 2% lidocaine and 1.2 ml normal saline, for a total volume of 3 ml per arm) was used for subjects with thicker skin. | **MFU-V:** Ulthera® System (Ulthera, Inc., Raleigh, NC, USA)  **CaHA-CMC:** Radiesse®, Merz North America, Inc. | - Arm visual analogue scale  - Skin firmness (R0) and elasticity (R2),  - Clinician and patients global aesthetic improvement,  - Patients’ satisfaction,  - Adverse events |
| Smith, 2020 ^6^ | USA | Interventional (Pre-Post) | 90 d | 60 | F (100) | With skin laxity on the outer thighs with a body mass index less than 28 kg/m^2^. | 46.9  (30-59) | Caucasian, Hispanic, African American, Asian | Outer Thighs | **MFU-V:** A single treatment using a 4-MHz transducer at a focal depth of 4.5 mm and a 7-MHz transducer at a focal depth of 3.0 mm, delivering a total of 75 lines per transducer per outer thigh.  **CaHA-CMC:** Immediately following MFU-V treatment. Treatment received with 1.5 mL of CaHA-CMC diluted 1:1 with 1.5 mL of 2% lidocaine solution per outer thigh. | **MFU-V:** Ulthera® System (Ulthera, Inc., Raleigh, NC, USA)  **CaHA-CMC:** Radiesse® ((Merz North America, Inc., Raleigh, NC, USA) | - Body image,  - Appraisal of excess skin,  - Patients’ satisfaction,  - Appearance-related psychosocial  distress,  - Adverse events. |
| Casabona, 2014 ^7^ | Brazil | Histological findings | 60 d | 1 | F (100) | Healthy. | 45 years | NM | Thigh | **CaHA-CMC:** Injection with filler from the subcutaneous plane to the superficial dermis until a papula was seen.  **MFU-V:** Immediately following injection. A single treatment using 3 transducers at depth of 4.5 mm, 3.0 mm, and 1.5 mm, providing a total of 30 shots (10 per each transducer) | **CaHA-CMC:** NM  **MFU-V:** Ulthera (No more information) | - Neocollagenesis |
| Casabona, 2017 ^8^ | Brazil | Observational (Retrospective) | 90 d | 20 | F (100) | With a moderate-to-severe cellulite and body mass index less than 25 kg/m^2^. | 40±8.2 | NM | Buttocks and thighs | **MFU-V:** MFU-V was directed using a 4 MHz transducer at a focal depth of 4.5 mm and a 7 MHz transducer at a depth of 3.0 mm, delivering 75 lines per transducer for each side (25 lines per buttock or thigh treatment site).  **CaHA-CMC:** Immediately after MFU-V treatment. Treatment received with 1.5 ml CaHA-CMC diluted 1:1 with 1.5 ml of 2% lidocaine solution. | **MFU-V:** Ulthera® System (Ulthera, Inc., Raleigh, NC, USA)  **CaHA-CMC:** Radiesse® (Merz North America, Inc., Raleigh, NC, USA) | - Clinician skin laxity and cellulite improvement,  - Patients’ satisfaction,  - Adverse events and pain. |
|  |  | Histological findings | 90 d | 1 | F | With a moderate-to-severe cellulite and body mass index less than 25 kg/m^2^. | NM | NM | Thigh | **CaHA-CMC:** 6 different dilutions of CaHA-CMC diluted with 2% lidocaine (1:0.16, 1:0.3, 1:0.6, 1:1, 1:2, and 1:6.5). Each inner thigh was injected with 0.3 ml of each dilution. The left thigh (Control) only received CaHA-CMC treatment.  **MFU-V:** On the right thigh, each CaHA-CMC-treated square and 1 non–CaHA-CMC-treated area also received treatment with the MFU-V 4 MHz transducer(4.5 mm) and 7 MHz transducer (3.0 mm) at 5 lines per square for each transducer. | **CaHA-CMC:** Radiesse® (Merz North America, Inc., Raleigh, NC, USA)  **MFU-V:** Ulthera® System (Ulthera, Inc., Raleigh, NC, USA) | - Neocollagenesis. |
| Lim, 2021 ^9^ | Singapore | Interventional  (Pre-Post) | 6 m | 10  (Abdomen (n=6), back (n=1), thighs (n=2), legs (n=1)) | F (100) | Healthy with Striae Distensae Albae | 36 (27-58) | Asian (Chinese and Malay) | Abdomen, back, thigh, leg | **MFU-V:** 3 transducers with a frequency of either 4, 7, or 10 MHz, and a focal depth of either 4.5, 3.0, and 1.5 mm, delivering a total of 480–880 lines. 200–400 lines were delivered at a depth of 4.5 mm, 200–400 lines were delivered at a depth of 3.0 mm, and 80 lines were delivered at a depth of 1.5 mm.  **CaHA-CMC:** Immediately following MFU-V treatment. CaHA-CMC+ was diluted with normal saline in a ratio of 1:1. Depending on the surface area of the Striae Distensae Albae, a total of 3–6 mL of diluted CaHA-CMC+ were injected subdermally. | **MFU-V:** Ulthera® System (Ulthera, Inc., Raleigh, NC, USA)  **CaHA-CMC:** Radiesse®  (+) ((Merz North America, Inc., Raleigh, NC, USA) | - Striae Distensae Albae improvement,  - Striae Distensae Albae condition change,  - Patient self-assessment of striae distensae albae,  - Patient global aesthetic improvement scale,  - Patient satisfaction,  - Adverse events. |
| Juhász, 2023 ^10^ | USA | Interventional  (Pre-Post) | 24 w | 20 | F (100) | With moderate-to-severe skin laxity of the anterior thigh and knee | 40-71 | Caucasian, Black | Knee | **MFU-V:** Dual-depth MFU-V using a 7MHz (3.0 mm) and a 10 MHz (1.5mm) or triple-depth treatment using a 4 mHz (4.5 mm), a 7MHz (3.0 mm), and a 10MHz (1.5mm) transducer if the 4.5 mm aponeurotic band was visualized, otherwise they were also treated with dual-depth therapy. Of the subjects receiving dual-depth MFU-V, an average of 173 lines of 3 mm depth and 59 lines of 1.5mm depth were used.  for subjects receiving triple-depth MFU-V, an average of 108 lines of 4.5mm depth, 100 lines of 3 mm depth, and 45 lines of 1.5mm depth were given. All subjects received a total average of 240 lines (range: 127–381) of MFU-V treatment at baseline.  **CaHA-CMC:** Immediately after MFU-V treatment. Intradermal injection of 1:1 diluted CaHA-CMC. An average of 2.2 mL of diluted CaHA-CMC was injected at baseline. Two subjects received an additional average of 2.2 mL of diluted CaHA-CMC at Week 6. | **MFU-V:** Ulthera® System (Ulthera, Inc., Raleigh, NC, USA)  **CaHA-CMC:** Radiesse® ((Merz North America, Inc., Raleigh, NC, USA) | - Clinician and patient global aesthetic improvement scale,  - Patients’ satisfaction,  - Skin roughness index and rhytid depth (n=10),  - Adverse events. |
|  |  | Interventional  (Controlled) | 24 w | 20  (Control: 20 untreated knees of the patients) | F (100) | With moderate-to-severe skin laxity of the anterior thigh and knee | 40-71 | Caucasian, Black | Knee | **MFU-V:** Dual-depth MFU-V using a 7MHz (3.0 mm) and a 10 MHz (1.5mm) or triple-depth treatment using a 4 mHz (4.5 mm), a 7MHz (3.0 mm), and a 10MHz (1.5mm) transducer if the 4.5 mm aponeurotic band was visualized, otherwise they were also treated with dual-depth therapy. Of the subjects receiving dual-depth MFU-V, an average of 173 lines of 3 mm depth and 59 lines of 1.5mm depth were used.  for subjects receiving triple-depth MFU-V, an average of 108 lines of 4.5mm depth, 100 lines of 3 mm depth, and 45 lines of 1.5mm depth were given. All subjects received a total average of 240 lines (range: 127–381) of MFU-V treatment at baseline.  **CaHA-CMC:** Immediately after MFU-V treatment. Intradermal injection of 1:1 diluted CaHA-CMC. An average of 2.2 mL of diluted CaHA-CMC was injected at baseline. Two subjects received an additional average of 2.2 mL of diluted CaHA-CMC at Week 6. | **MFU-V:** Ulthera® System (Ulthera, Inc., Raleigh, NC, USA)  **CaHA-CMC:** Radiesse® (Merz North America, Inc., Raleigh, NC, USA) | - Merz Scale grades,  - Dermal thickness. |
| Casabona, 2022 ^11^ | Spain | Case-series | 3-6 m | 2 | F (100) | **Subject 1:** Premenopausal woman presented with Grade 2 At Rest and Grade 3 hand-to-elbow Dynamic chest rhytids.  **Subject 2:** Post-menopausal woman presented with Grade 3 At Rest and Grade 3 hand-to-elbow Dynamic chest rhytids. | **Subject 1:** 51 year-old.  **Subject 2:** 60-year-old. | NM | Chest | ***Subject 1:***  **MFU-V:** A single treatment using 3 transducers, 4.5 mm, 3.0 mm, and 1.5 mm, delivering 100, 100, and 60 lines, respectively.  **CaHA-CMC:** Immediately after MFU-V treatment. Two syringes of CaHA-CMC diluted 1:1, 0.1 mL per trace at three entrance points. After 3 months, two syringes of CaHA-CMC diluted 1:1, 0.2 mL per trace at three entrance points.  ***Subject 2:***  **MFU-V:** A single treatment using 3 transducers, 4.5 mm, 3.0 mm, and 1.5 mm, delivering 100, 100 lines per each transducer.  **CaHA-CMC:** Immediately after MFU-V treatment. Two 1.5 mL syringes of CaHA-CMC diluted 1:1, 0.2 mL per trace at three entrance points. After 3 months, two 1.5 mL syringes of CaHA-CMC diluted 1:1, 0.2 mL per trace at three entrance points. | **MFU-V: Ulthera® System (Ulthera, Inc., Raleigh, NC, USA)**  **CaHA-CMC:** Radiesse® (Merz North America, Inc., Raleigh, NC, USA) | Gluteus Laxity Scale |
|  |  |  |  | 2 | F (100) | **Subject 1:** Pre-menopausal woman with a body mass index of 19 kg/m^2^ presented with a score of Grade 3 At Rest on the validated Gluteus Laxity Scale.  **Subject 2:** Pre-menopausal woman with a BMI < 24 kg/ m^2^, normal gluteus volume with a score of Grade 3 At Rest on the Gluteus Laxity Scale. | **Subject 1:** 38-year-old.  **Subject 2:** 39-year-old. | NM | Buttocks | ***Subject 1:***  **MFU-V:** A single treatment using 3 transducers,  4.5 mm, 3.0 mm, 1.5 mm, delivering 550, 550, and 440 lines.  **CaHA-CMC:** Immediately after MFU-V treatment. Five 1.5 mL syringes of CaHA-CMC diluted 1:1, 0.2 mL per trace at three entrance points. After 3 months, two 1.5 mL syringes of CaHA-CMC diluted 1:1, 0.2 mL per trace at three entrance points.  After 6 months, two additional 1.5 mL two additional syringes of CaHA-CMC with lidocaine.  ***Subject 2:***  **MFU-V:** A single treatment using 3 transducers, 4.5 mm, 3.0 mm, and1.5 mm, delivering360, 360, 240 lines.  **CaHA-CMC:** Immediately after MFU-V treatment. one 1.5 mL syringe of CaHA-CMC dilute 1:1 per side and four 1.5 mL syringes of CaHA-CMC diluted 1:2 per side at four entrance points. After 3 months, one 1.5 mL CaHA-CMC diluted 1:1 per side and four 1.5 mL syringes of CaHA-CMC diluted 1:2 per side at four entrance points. | **MFU-V: Ulthera® System (Ulthera, Inc., Raleigh, NC, USA)**  **CaHA-CMC:** Radiesse® (Merz North America, Inc., Raleigh, NC, USA) | Gluteus Laxity Scale |

^1^Age is reported either in mean and standard deviation (SD), mean (minimum-maximum), or minimum-maximum. ^*^Mean age in the total population was only reported.

MFU-V: Microbicides ultrasound with visualization; CaHA-CMC: Calcium hydroxylapatite; F: Female; B: Both; d: Day; w: Week; m: Month.

**Supplemental Table 3.** Risk of bias according to Risk Of Bias In Non-randomized Studies (ROBINS-I).

| **Author, year** | **Pre-intervention** | | **At intervention** | **Post-intervention** | | | |
| --- | --- | --- | --- | --- | --- | --- | --- |
|  | **Bias due to/in** | | **Bias in** | **Bias due to/in** | | | |
|  | **Confounding** | **Selection of participants** | **Classification of intervention** | **Deviations from intended interventions** | **Missing data** | **Measurement of outcomes** | **Selection of the reported result** |
| Kerscher, 2019 ^1^ | Serious Risk | Serious Risk | Low Risk | Low Risk | Low Risk | Low Risk | Low Risk |
| Casabona, 2017 ^2^ | Serious Risk | Low Risk | Low Risk | Low Risk | Low Risk | Serious Risk | Low Risk |
| Casabona, 2017 ^3^ | Serious Risk | Low Risk | Low Risk | Low Risk | Low Risk | Serious Risk | Low Risk |
| Yutskovskaya, 2020 ^4^ | Serious Risk | Low Risk | Low Risk | Low Risk | Low Risk | Serious Risk | Low Risk |
| Ramirez, 2021 ^5^ | Serious Risk | Low Risk | Low Risk | Low Risk | Low Risk | Serious Risk | Low Risk |
| Smith, 2020 ^6^ | Serious Risk | Low Risk | Low Risk | Low Risk | Low Risk | Serious Risk | Low Risk |
| Casabona, 2017 ^8^ | Serious Risk | Low Risk | Low Risk | Low Risk | Low Risk | Serious Risk | Low Risk |
| Lim, 2021 ^9^ | Serious Risk | Low Risk | Low Risk | Low Risk | Low Risk | Serious Risk | Low Risk |
| Juhász, 2023 ^10^ | Serious Risk | Low Risk | Low Risk | Low Risk | Low Risk | Serious Risk | Low Risk |

**Supplemental Table 4.**NIH quality assessment tool for Case series studies

|  | Casabona, 2022 ^11^ |
| --- | --- |
| **Was the study question or objective clearly stated?** | Y |
| **Was the study population clearly and fully described, including a case definition?** | Y |
| **Were the cases consecutive?** | NA |
| **Were the subjects comparable?** | N |
| **Was the intervention clearly described?** | Y |
| **Were the outcome measures clearly defined, valid, reliable, and implemented consistently across all study participants?** | Y |
| **Was the length of follow-up adequate?** | Y |
| **Were the statistical methods well-described?** | NA |
| **Were the results well-described?** | Y |
| **Quality Rating (Good, Fair, or Poor)** | Fair |

Y: Yes; N: No; CD: cannot determine; NA: not applicable; NR: not reported

**Supplemental Table 5.**Findings of the aesthetic effectiveness and skin quality for combined MFU-V and CaHA-CMC treatment.

| **Author, year** | **Treated region** | **Outcome/ Assessment method** | **Findings** |
| --- | --- | --- | --- |
| Kerscher, 2019 ^1^ | Jawline | - **Clinician sagging improvement scale**/ Merz Aesthetic Scales (4 as very sever, 0 as no skin sagging)  - **Clinician global aesthetic**  **improvement scale**/ GAIS (1 as very much improved, 5 as worse)  - **Skin laxity and jawline contour**/ Standardized photographic documentation  - **Skin thickness and firmness**/ Cutometry | - **Clinician sagging improvement scale**: Proportion of subjects with improvement of at least one point was observed to increase upon implementing combined MFU-V and CaHA-CMC treatment.  At week 4 (MFU-V only): 33%;  At week 8 (MFU-V only): 56%;  At week 24 (Combined): 78%;  At week 48 (Combined): 89%.  - **Clinician global aesthetic improvement scale**: Further improvement in the GAIS score was reported after 24 and 48 weeks, with the majority of subjects rated as much or very much improved.  *Frontal view:* At week 12 (MFU-V only): Improved=50%, Much improved=50%; at week 24 (Combined): Improved=22%, much improved=78%; at week 48 (Combined): Much improved=50%, very much improved= 50%.  *Side view:* At week 12 (MFU-V only): Improved=50%, Much improved=50%; at week 24 (Combined): Improved=11%, Much improved=78%, very much improved= 11%; at week 48 (Combined): Improved=17%, Much improved=33%, Very much improved= 50%.  - **Skin laxity and jawline contour**: Standardized photography of all subjects demonstrated improvements in skin laxity and the jawline contour following treatments with MFU-V and CaHA-CMC, with results lasting over 48 weeks.  - **Skin thickness and firmness**: In subjects who received combined treatment, skin thickness increased significantly (p<0.05) in the jawline area by up to 1834 µmol at week 24 compared to the baseline, while skin firmness improved significantly (p<0.05) at week 48.  *Thickness:* At baseline: 1651.056 µmol; at week 4 (MFU-V only): 1822.278; at week 12 (MFU-V only): 1776.722; at week 24 (Combined): 1834.167 (p<0.05 vs baseline); at week 48 (Combined): 1683.250.  *Firmness:* At baseline: 0.079 mmol; at week 4 (MFU-V only): 0.113; at week 12 (MFU-V only): 0.077; at week 24 (Combined): 0.102; at week 48 (Combined): 0.100 (p<0.05 vs baseline). |
| Casabona, 2017 ^2^ | Both sides of the face | - **Scar severity**/ Goodman Acne Scar Scale  - **Patients’ satisfaction**/ 5-point scale (very unsatisfied to very satisfied) | - **Scar severity**: A significant overall improvement in baseline acne scar severity at day 90 was reported.  *Overall mean±SD acne scar scores:* At baseline 7.6±1.7, at day 90: 3.8±0.9, p=0.002.  *By severity of scars:*  Subjects with mild scars: At baseline: 1.0±0.5, at day 90: 0.7±0.5, p= 0.250.  Subjects with sever scars: At baseline: 2.8±1.8 to 1.1±1.5, p= 0.063.  A clear but statistically insignificant trend was apparent with the greatest improvement observed among subjects with the greatest scar severity.  - **Patients’ satisfaction**: At day 90 (Mean±SD): 4.9±0.3. Subjects were very satisfied (n=9) or satisfied (n=1) with the aesthetic at the end of study. |
| Casabona, 2017 ^3^ | Neck and/or decolletage | - **Clinician** **neckline score**/ Allergan transverse neck lines scale  - **Clinician** **decolletage score**/ Merz Aesthetics Décolleté wrinkles at rest scale, Fabi-Bolton chest wrinkle scale  - **Patients’ satisfaction**/ NM | - **Neckline score**: Approximately 20% of subjects had very severe horizontal necklines, 25% had severe, and about 55% had moderate lines. After treatment, in over two-thirds of subjects, lines were then classed as mild, and around 30% had moderate lines. Only one subject was rated as having severe horizontal necklines, and no subjects had very severe lines.  At baseline: Mean score of 2.6 (moderate-to-severe lines);  At day 90: 1.3 (mild lines) (p<0.001 vs baseline).  - **Decolletage score**:  At baseline: mean of 2.6 on the Merz Aesthetics and 3.3 on the Fabi-Bolton scales (both indicating moderate lines due to the one-point score difference between the scales);  At day 90: 1.1 and 1.8, respectively (p<0.001 vs baseline).  - **Patients’ satisfaction**:  At baseline (Mean±SD): 3.2±0.6 for the neck and 3.1±0.6 for the decolletage, indicating patients were neither satisfied nor dissatisfied;  At day 90: 4.5±0.6 and 4.4±0.6, respectively, indicating patients were satisfied to very satisfied with the appearance of their neck and decolletage;  Combined satisfaction scores for the neck and décolletage:  At baseline: 3.2±0.6;  At day 90: 4.5±0.6 (p<0.001 vs baseline). |
| Yutskovskaya, 2020 ^4^ | Face, neck, décolleté | - **Clinician age-related aesthetics improvement**/ Merz Aesthetics Scales  - **Volumes of the submental area and a cervicomandibular** **angle**/ 3D Models of patients  - **Clinician and patients global aesthetic improvement**/ GAIS | - **Clinician age-related aesthetics improvement**: the severity of age-related changes based on the Merz Aesthetics Scales demonstrated improvement in all treated areas versus baseline as assessed by the investigator.  At baseline (Mean±SD): Marionette lines score: 2.47 ± 0.8, jawline contour score: 2.2 ± 0.7, neck score: 2.1 ± 0.7 points;  At month 15 (Mean±SD): Marionette lines score: 1.8 ± 0.7 (p≤0.00003 vs baseline), jawline contour score: 1.89 ± 0.56 (p≤0.005 vs baseline), neck score: 1.7 ± 0.6 (p≤0.005 vs baseline) points.  - **Volumes of the submental area and a cervicomandibular** **angle**:  *Submental volume:* The volume changes varied from 0.4 to 24.1 cm^3^; negative changes were observed only in one patient (an increase by 3.9 cm^3^).  *Cervicomandibular angle:* Changes varied from 0 to 8 degrees; negative changes were observed only in one patient (an increase in the angle by 14 degrees).  - **Clinician and patients global aesthetic improvement**:  At month 8:  Investigators: Right Side (CaHA-CMC+MFU-V): 2.00 ± 0.57.  Patients: Right Side (CaHA-CMC+MFU-V): 1.70 ± 0.90.  At month 15:  Investigators: Left Side (MFU-V+CaHA-CMC): 3.00 ± 0.80; Right Side (CaHA-CMC+MFU-V): 2.78 ± 0.80.  Patients: Left Side (MFU-V+CaHA-CMC): 2.47 ± 1.00; Right Side (CaHA-CMC+MFU-V): 2.40 ± 1.00. |
| Ramirez, 2021 ^5^ | Brachial regions | - **Clinician arm visual analogue scale**/ VAS for upper arms (Type I = no laxity, to Type V = very severe laxity).  - **Skin firmness (R0) and elasticity (R2)**/ cutometer (lower R0 value denotes firmer skin, higher R2 denotes more elastic skin)  - **Clinician and patients global aesthetic improvement scale**/ GAIS  - **Patients’ satisfaction**/ Subject global satisfaction scale (1 as very dissatisfied to 5 as very satisfied). | - **Clinician arm visual analogue scale**: Pre- and post- treatment photographs showed the improvement in overall skin quality and laxity from baseline over the study period (p<0.05).  At baseline (Mean±SD): 2.9±0.6;  At week 4 (Mean±SD): 2.5±0.6 (p=0.0192 vs baseline);  At week 12 (Mean±SD): 2.1±0.7 (p=0.0015 vs baseline);  At week 24 (Mean±SD): 2.3±0.8 (p=0.0006 vs baseline).  - **Skin firmness (R0) and elasticity (R2)**:  *Skin firmness:* A decrease in the mean R0 was reported (the lower the R0, the better the firmness of the skin) throughout the course of the study. Statistically significant improvements were observed from 12 weeks onwards.  At baseline (Mean±SD): 0.515±0.098 mm;  At week 4 (Mean±SD): 0.496±0.075 mm (p=0.4954 vs baseline);  At week 12 (Mean±SD): 0.463±0.071 mm (p=0.0362 vs baseline);  At week 24 (Mean±SD): 0.433±0.049 mm (p=0.0013 vs baseline).  *Skin elasticity:* The mean R2 reading (closer to 1, the better the elasticity of the skin) improved significantly from baseline at all time points.  At baseline (Mean±SD): 0.816±0.032;  At week 4 (Mean±SD): 0.833±0.034 (p=0.0007 vs baseline);  At week 12 (Mean±SD): 0.847±0.037 (p=0.0012 vs baseline);  At week 24 (Mean±SD): 0.841±0.018 (p=0.0003 vs baseline).  - **Clinician global aesthetic improvement scale**:  *Clinicians*: Based on investigator's ratings, over 70% of all subjects showed an improved aesthetic appearance compared with baseline at all time- points throughout the study.  At week 4: Out of 11 patients, 3 had no change, 6 was scored to have improved, and 2 much improved;  At week 12: Out of 8 patients, 1 had no change, 4 was scored to have improved, and 3 much improved;  At week 24: Out of 12 patients, 2 had no change, 5 was scored to have improved, 3 much improved, and 2 very much improved.  *Patients*: Over 37% of subjects were reported to have much improved and very much improved brachial laxity by 12 and 24 weeks. There were no reported cases with worsening of brachial laxity  - **Patients’ satisfaction**:  At week 4: Out of 11 patients, 5 were neutral, 4 were satisfied, and 2 very satisfied;  At week 12: Out of 8 patients, 2 were neutral, and 6 were satisfied;  At week 24: Out of 12 patients, 5 were neutral, 5 were satisfied, and 2 very satisfied. |
| Smith, 2020 ^6^ | Outer Thighs | - **Body image**/ BODY-Q; ranges from 7 to 28 (with a high score indicating that a patient feels more positive about their body image)  - **Appraisal of excess skin**/ BODY-Q; ranges from 7 to 28 (with a high score indicating that a patient feels less bothered by their excess skin)  - **Patients’ satisfaction**/ BODY-Q; Satisfaction with Hips and Outer Thighs Scale ranges from 5 to 20 (with a high score indicating that a patient feels more).  - **Appearance-related psychosocial distress**/ BODY-Q; ranges from 8 to 32 (with a low score indicating that a patient feels less distressed by their appearance). | - **Body image**: Patients reported feeling more positive about their body image.  At baseline (Mean±SD): 12.9±4.0;  At day 90 (Mean±SD): 19.9±4.2 (p<0.01 vs baseline).  - **Appraisal of excess skin**: Patients reported feeling less bothered by their excess skin.  At baseline (Mean±SD): 14.7±5.5;  At day 90 (Mean±SD): 20.5±5.8 (p<0.01 vs baseline).  - **Patients’ satisfaction**: Patients reported satisfaction with Hips and Outer Thighs.  At baseline (Mean±SD): 7.9±2.4;  At day 90 (Mean±SD): 12.8±3.6 (p<0.01 vs baseline).  - **Appearance-related psychosocial distress**: patient felt less distressed by their appearance.  At baseline (Mean±SD): 17.0±5.1;  At day 90 (Mean±SD): 10.9±3.6 (p<0.01 vs baseline). |
| Casabona, 2017 ^8^ | Buttocks and thighs | - **Clinician skin laxity and cellulite improvement**/ Cellulite severity scale (CSS) including (1) number of evident depressions, (2) depth of depressions, (3) morphologic appearance of skin surface alterations, (4) grade of laxity, flaccidity or sagging skin, and (5) the classification scale originally described by Nürnberger and Müller.  - **Patients’ satisfaction**/ NM | - **Clinician skin laxity and cellulite improvement**:  *Skin depressions:*  At baseline: 75% of the patients were evaluated as moderate-to-severe skin depressions. Depression depth was rated as superficial in 40% of cases and medium-depth to deep in 60%.  At day 90: 4 women had no evident depressions, and 11 had only a small number of depressions compared with 0 and 5 women, respectively, at baseline. The depth of depressions was also decreased, with 80% of the women having no depressions or depressions of superficial depth.  *Morphologic appearance of skin surface alterations:*  At baseline: Appearance was described as “orange peel” in 8 women, “cottage cheese” in 5 women, and “mattress-like” in 5 women.  At day 90: Skin surface appearance was improved, with 11 women having no raised areas compared with 2 at baseline. An “orange peel” appearance was recorded for 8 women at baseline but only 4 at day 90. A “mattress-like” appearance was reported in 5 women at baseline.  *Laxity, flaccidity or sagging skin:*  At baseline: Evaluators graded 50% of the women as having slight laxity, flaccidity, or sagging, and 50% as having moderate-to-severe laxity.  At day 90: Overall, 8 women showed no laxity, flaccidity, or sagging skin at day 90 compared with 0 at baseline. A further 9 or 10 women were rated as having slight skin laxity by evaluator 1 and evaluator 2, respectively.  *Nürnberger and Müller Classification scale:*  At baseline: Evaluators were in agreement when grading women with the Nürnberger and Müller classification, with 90% of the women rated as grade 2 or 3.  At day 90: Evaluators agreed on improvements in the Nürnberger and Müller classification, with 12 subjects having a grade 1 classification compared with only 2 at baseline.  *Overall score:* Evaluators reported statistically significant improvements for each CSS item compared with baseline.  At 90 days: Mean improvement in the overall score of 4.5 (p< 0.001 vs baseline).  - **Patients’ satisfaction**: After treatment 10 patients (50%) were very satisfied and 9 women (45%) were satisfied. |
| Lim, 2021 ^9^ | Abdomen, back, thigh, leg | - **Clinician striae distensae albae (SDA) improvement**/ 5-point scale (0 as no change to 4 as excellent improvement).  - **Clinician striae distensae albae score**/ Including (1) maximum width, (2) appearance in terms of color mismatch, (3) finish (matt or shiny), (4) contour.  - **Patient self-assessment of striae distensae albae**/ 10-point scale (1 as not bothered to 10 as significantly bothered).  - **Patient global aesthetic improvement scale**/ GAIS  - **Patient satisfaction**/ 5-point scale (1 as very unsatisfied and 5 as very satisfied). | - **Clinician striae distensae albae improvement**: SDA was improved in all patients at 3 months and greater improvements at 6 months.  At 3 months: All patients had at least a mild improvement (1%–25%);  At 6 months: 8 patients had a moderate to good improvement (25%–50%), 2 had a good improvement (51%–75%).  - **Clinician striae distensae albae score**: SDA score showed improvement over time.  At baseline: Mean score of 11.6;  At month 1: Mean score 11.1 (p>0.05 vs baseline);  At month 3: Mean score of 7.9 (p=0.005 vs baseline);  At month 6: Mean of 6.2 (p=0.005 vs baseline).  - **Patient self-assessment of striae distensae albae**: At the end of the study, patient score was reduced by 1–6 points, with a mean reduction of 2.7 points from 7.0 at baseline to 4.3 at 6 months;  At month 1: 2 patients were less bothered with their SDA than they were at baseline;  At month 3: 7 patients were less bothered with their SDA than they were at baseline;  At month 6: All patients recorded improvement in their SDA.  - **Patient global aesthetic improvement scale**:  At month 1: Out of 10, 3 patients reported improvement;  At month 3: All patients reported improvement, 2 of whom recorded as much improved;  At month 6: Out of 10, 3 patients had improved, 4 had much improved, and 3 had very much improved records.  - **Patient satisfaction**:  At month 3: Out of 10, 8 patients were satisfied with the results.  At month 6: All patients were satisfied (5 patients) or very satisfied (5 patients) with the treatment. |
| Juhász, 2023 ^10^ | Knee | - **Clinician and patient global aesthetic improvement scale**/ GAIS  - **Patients’ satisfaction**/ a 10-point scale  - **Skin roughness index and rhytid depth** (a subset of 10 patients)/ 3D-imaging | - **Clinician and patient global aesthetic improvement scale**:  Clinician (Mean±SD):  At week 6: 2.8±0.6 (mild improvement);  At week 12: 2.8±0.7 (mild to moderate improvement);  At week 24: 3.2±0.7 (mild improvement).  Patients (Mean±SD):  At week 6: 3.1±0.9 (mild to moderate improvement);  At week 12: 2.7±0.8 (mild to moderate improvement);  At week 24: 3.1±1.0 (mild improvement).  - **Patients’ satisfaction**:  At week 6 (Mean±SD): 5.7±2.2;  At week 12 (Mean±SD): 6.6±2.2;  At week 24 (Mean±SD): 6.1±2.9.  - **Rhytid depth and skin roughness** (a subset of 10 patients):  *wrinkle depth:*  At baseline (Mean±SD): 0.21±0.04 mm;  At week 12 (Mean±SD): 0.20±0.05 mm (p=0.36 vs baseline)  *Skin roughness index:*  At baseline (Mean±SD): 0.66±0.24;  At week 12 (Mean±SD): 0.60±0.29 (p=0.25 vs baseline). |
|  |  | - **Aesthetics improvement**/ Merz Scale  - **Dermal thickness**/ Tissue attenuation coefficient as a surrogate for dermal thickness; Optical coherence tomography (OCT; Beckman Laser Institute, Irvine, CA) | - **Aesthetics improvement**:  At baseline (Mean±SD): No significant difference between the Merz Scale grades of the treated and untreated knees.  Treated knee: 2.7±0.6;  Untreated knee: 2.6±0.5 (between group p>0.05).  At week 12 (Mean±SD): Merz Scale grades for the treated knee significantly improved.  Treated knee: 1.9±0.7 (p=0.001063 vs baseline);  Untreated knee: 2.3±0.7 (p>0.05 vs baseline).  At week 24 (Mean±SD): Merz Scale grades for the treated knee significantly improved.  Treated knee: 2.0±0.8 (p=0.004423 vs baseline);  Untreated knee: 2.3±0.7 (p>0.05 vs baseline).  When analyzing dual- and triple-depth MFU-V treatment groups individually, there was no significant difference from baseline to Week 12 or 24 for either group.  - **Dermal thickness**:  At baseline (Mean±SD):  Treated knee: Average of 1.98±0.36;  Untreated knee: average of 2.00±0.40  At Week 12 (Mean±SD):  Treated knee: 2.04±0.38 (p=0.36 vs baseline);  Untreated knee: 2.00±0.33 (p=0.93 vs baseline).  At week 24 (Mean±SD):  Treated knee: 1.86±0.30 (p=0.75 vs baseline);  Untreated knee: 1.97±0.42 (p=0.23 vs baseline). |
| Casabona, 2022 ^11^ | Chest | **Laxity _ Chest**/ Gluteus Laxity Scale at rest and dynamic | **Laxity _ Chest**  *Subject 1:* Presented with Grade 2 At Rest and Grade 3 Dynamic chest rhytids. Three months after the final CaHA-CMC treatment, chest appearance improved to Grade 1 At Rest and Dynamic rhytids.  *Subject 2:* Presented with Grade 3 At Rest and Grade 3 Dynamic chest rhytids. Three months after the final CaHA-CMC treatment, chest appearance improved to Grade 1 At Rest and Dynamic rhytids |
|  | Buttocks | **Laxity _ Buttocks**/ Gluteus Laxity Scale at rest and dynamic | **Laxity _ Buttocks**  *Subject 1:* Presented with a Grade 3 At Rest score on the Gluteus Laxity Scale. Three months after her final CaHA-CMC treatment, patient achieved a Grade 1 At Rest score  *Subject 2:* Presented with a Grade 3 At Rest score on the Gluteus Laxity Scale. Three months after her final CaHA-CMC treatment, patient achieved a Grade 1 At Rest score |

**Supplemental Table 6.**Histological findings of the mechanism of action for combined MFU-V and CaHA-CMC treatment.

| **Author, year** | **Outcome/ Assessment method** | **Findings^1^** |
| --- | --- | --- |
| Yutskovskaya, 2020 ^4^ | **- Neocollagenesis**/ Immunohistochemical staining  **- Angiogenesis**/ Immunohistochemical staining  **- Elastogenesis**/ Van Gieson’s picrofuchsin and Weigert’s fuchsin stain  **- CD34**/ Immunohistochemical staining  **- Proliferation** **index/** Immunohistochemical staining | **- Neocollagenesis**: A significant increase of collagen type I and collagen type III after CaHA-CMC injection which continued to increase after the combined treatment (biopsies performed at visit month 12, 4 months after admiration of MFU-V, were used for evaluation of the effect of stimulation with CaHA-CMC and MFU-V).  *Collagen type I:*  After CaHA-CMC only (Mean±SD): 0.9±0.5–2.4±0.3 points (p<0.05 vs baseline);  After combined treatment: up to 4.4±0.3 (p<0.05 vs baseline).  *Collagen type III:*  After CaHA-CMC only (Mean±SD): 1.5±0.2–3.5±0.3 points (p<0.05 vs baseline);  After combined treatment: 5.7±0.2 points (p<0.05 vs baseline).  **- Angiogenesis**: A significant increase after CaHA-CMC injection which continued to increase after the combined treatment.  After CaHA-CMC only (Mean±SD): 1 – 2 points (p<0.05 vs baseline);  After combined treatment: up to 5.2±0.2 points (p<0.05 vs baseline).  **- Elastogenesis**: A significant increase after CaHA-CMC injection which continued to increase after the combined treatment.  After CaHA-CMC only (Mean±SD): 1 – 3.6±0.2 points (p<0.05 vs baseline);  After combined treatment: up to 5.5±0.2 points (p<0.05 vs baseline).  **- CD34**: A significant increase of CD34, an endothelial marker for newly formed blood vessels, after the injections of CaHA-CMC which continued to increase after MFU-V treatment.  After CaHA-CMC only (Mean±SD): 0.10±0.07–2.6 ±0.4 points (p<0.05 vs baseline);  After combined treatment: up to 5.4±0.2 points (p<0.05 vs baseline).  **- Proliferation** **index**: Ki67 expression was increased after CaHA-CMC injection, primarily due to neoangiogenesis, and relatively decreased after the MFU-V procedure.  After CaHA-CMC only (Mean±SD): 0.80±0.08–2.6±0.2 points (p<0.05 vs baseline);  After combined treatment: decreased up to 1.6±0.2 points (p>0.05 vs baseline). |
| Casabona, 2014 ^7^ | **- Neocollagenesis**/ Verhoeff's Van Gieson stain and Masson's trichrome stain | **Neocollagenesis:** Findings showed more thickening and dense fibers of collagen, proving that there were an enhancement of the number and quality of collagen and elastin fibers in the combination treatment site compared to MFU-V alone. |
| Casabona, 2017 ^8^ | **- Neocollagenesis**/ Picrosirius staining plus polarization microscopy | - **Neocollagenesis:**   At day 90: histologic analysis showed peak neocollagenesis in samples treated with the 1:1 dilution, whether with CaHA-CMC alone or in combination with MFU-V.  When CaHA-CMC was combined with MFU-V, the 1:1 dilution was associated with a 251% increase in the number of collagen type III fibers compared with control tissue.  Conversion of collagen type III into collagen type I: The highest conversion occurred in samples injected with 1:1 and 1:0.6 CaHA-CMC dilutions without subsequent MFU-V treatment compared to the untreated control tissue, with increases of 103% and 93%, respectively. Following the combine treatment, conversion to type I collagen was only slightly evident (increase of 41%). |

^1^Each histological result is based on one sample.

**Supplemental Table 7.** Findings of safety for combined MFU-V and CaHA-CMC treatment.

| **Author, year** | **Findings** |
| --- | --- |
| Kerscher, 2019 ^1^ | No unexpected adverse events were reported, and both treatments were well tolerated.  Slight erythema was seen in fairer skin phototypes immediately after MFU-V treatment, which lasted for approximately 30 to 60 min. This was an expected reaction for these subtypes. In all cases there was minimal or no downtime. Swelling and bruising were seen as injection-related side effects that resolved without sequalae within several days after CaHA-CMC filler injections. No serious adverse events were reported. |
| Casabona, 2017 ^2^ | There were no reports of adverse events. |
| Casabona, 2017 ^3^ | Mild pain was experienced by 90% of subjects during the procedure; 10% reported no discomfort at all. All subjects experienced bruising, which resolved in 3-7 days. No other adverse events were reported. |
| Yutskovskaya, 2020 ^4^ | Following CaHA-CMC injection at visit 1 (Day 1), patients graded pain intensity in the face as 2.00±0.90 points and as 1.30±0.58 points in visit 5 (Month 15). Pain intensity in the neck was graded 1.78±0.97 points at visit 1 and as 1.40±0.60 points at visit 5, and pain in the décolleté was 1.78±0.78 points and 1.47±0.60 points, respectively. For all treated areas, subjective pain intensity during procedures reduced by stage 5 of the study.  Erythema, ecchymoses, and petechiae at the injection site were reported, as well as occasional hematomas, which spontaneously resolved within a short time after injection. During the 15-month follow up, no further adverse events were reported. |
| Ramirez, 2021 ^5^ | No serious adverse events were reported. All adverse events were mild and transient in nature. The most common adverse events reported were mild bruising (92%; 11/12) and redness (25%; 3/12), which resolved spontaneously within a week. One subject reported mild paresthesia (electric shock sensations) down the left arm, which also resolved spontaneously over two weeks. |
| Smith, 2020 ^6^ | There were no cases of severe bruising or severe pain. However, patients did experience mild pain, mild bruising, mild erythema, and mild edema, all of which resolved in 2 to 3 days. |
| Casabona, 2017 ^8^ | The combination treatment was well tolerated, with no cases of severe bruising or severe pain. All patients reported mild pain the day after the procedures with a mean duration of 2 days. Mild bruising was observed in 18 women and had resolved within 1 week. Other mild events observed were erythema in 2 patients, edema in 10 patients, and injection-site induration in 5 patients, all of which resolved in 2‒3 days |
| Lim, 2021 ^9^ | No side effects were observed during the follow-up sessions. Some bruises were seen immediately after the filler injection in 4 patients, all of which resolved without treatment. |
| Juhász, 2023 ^10^ | At Week 6, 68 percent of patients reported an adverse events within the first month of treatment. All post-procedure adverse events were of mild intensity; including bruising (63%), swelling (52%), temporary palpable nodules (37%), erythema (32%), skin sensitivity (26%), local pain/discomfort (21%), and local numbness (5%). At Weeks 12 and 24, no adverse events were reported. |

**REFRENCES:**

1. Kerscher M, Nurrisyanti AT, Eiben-Nielson C, Hartmann S, Lambert-Baumann J. Clinical and Biophysical Outcomes of Combining Microfocused Ultrasound with Visualization and Calcium Hydroxylapatite Filler for Facial Treatment. *Dermatol Ther (Heidelb)*. Mar 2019;9(1):135-142. doi:<https://dx.doi.org/10.1007/s13555-018-0273-y>

2. Casabona G. Combined use of microfocused ultrasound and a calcium hydroxylapatite dermal filler for treating atrophic acne scars: A pilot study. *J Cosmet Laser Ther*. Oct 2018;20(5):301-306. doi:<https://dx.doi.org/10.1080/14764172.2017.1406606>

3. Casabona G, Nogueira Teixeira D. Microfocused ultrasound in combination with diluted calcium hydroxylapatite for improving skin laxity and the appearance of lines in the neck and decolletage. *J*. Feb 2018;17(1):66-72. doi:<https://dx.doi.org/10.1111/jocd.12475>

4. Yutskovskaya YA, Sergeeva AD, Kogan EA. Combination of Calcium Hydroxylapatite Diluted With Normal Saline and Microfocused Ultrasound With Visualization for Skin Tightening. Randomized Controlled Trial. *J Drugs Dermatol*. Apr 01 2020;19(4):405-411. doi:<https://dx.doi.org/10.36849/JDD.2020.4625>

5. Ramirez S, Puah IBK. Effectiveness of combined microfocused ultrasound with visualization and subdermal calcium hydroxyapatite injections for the management of brachial skin laxity. *J*. Dec 2021;20(12):3871-3879. doi:<https://dx.doi.org/10.1111/jocd.14573>

6. Smith JR, Sheehan M, Casas LA. Using the BODY-Q to Evaluate Appearance and Quality of Life Following Treatment of Skin Laxity of the Outer Thigh with Microfocused Ultrasound and Calcium Hydroxylapatite. Research Support, Non-U.S. Gov't. *Aesthet*. 10 24 2020;40(11):1219-1231. doi:<https://dx.doi.org/10.1093/asj/sjz372>

7. Casabona G, Michalany N. Microfocused ultrasound with visualization and fillers for increased neocollagenesis: clinical and histological evaluation. Case Reports. *Dermatol Surg*. Dec 2014;40 Suppl 12:S194-8. doi:<https://dx.doi.org/10.1097/DSS.0000000000000231>

8. Casabona G, Pereira G. Microfocused Ultrasound with Visualization and Calcium Hydroxylapatite for Improving Skin Laxity and Cellulite Appearance. *Plast*. Jul 2017;5(7):e1388. doi:<https://dx.doi.org/10.1097/GOX.0000000000001388>

9. Lim JTE. Treating Striae Distensae Albae in Asians: Efficacy and Safety of Combined MFU-V and CaHA. *Plast*. Feb 2021;9(2):e3429. doi:<https://dx.doi.org/10.1097/GOX.0000000000003429>

10. Juhasz M, Yale KL, Thatiparthi A, Babadjouni A, Mesinkovska NA. Combination of Microfocused Ultrasound with Visualization and Dilute Calcium Hydroxylapatite Filler for Moderate to Severe Knee Skin Laxity. *J Clin Aesthet Dermatol*. Feb 2023;16(2):14-18.

11. Casabona G. Combined Calcium Hydroxylapatite Plus Microfocused Ultrasound for Treating Skin Laxity of the Chest and Buttocks. *J Drugs Dermatol*. Jan 01 2022;21(1):27-30. doi:<https://dx.doi.org/10.36849/JDD.2022.6368>
